# A Simple Model of the Energy Harvester within a Linear and Hysteresis Approach

**DOI:** 10.3390/mi14020310

**Published:** 2023-01-25

**Authors:** Mikhail E. Semenov, Peter A. Meleshenko, Sergei V. Borzunov, Olga O. Reshetova, Andrey I. Barsukov

**Affiliations:** 1 Department of Digital Technologies, Voronezh State University, Universitetskaya sq. 1, 394018 Voronezh, Russia; 2 Department of Applied Mathematics and Mechanics, Voronezh State Technical University, XX-Letiya Oktyabrya st. 84, 394006 Voronezh, Russia; 3 Geophysical Survey of Russia Academy of Sciences, Lenina av. 189, 249035 Obninsk, Russia

**Keywords:** energy harvester, inverted pendulum, hysteresis, Preisach operator, Bouc–Wen model

## Abstract

In this article, a model of an energy harvester, the mechanical part of which is an inverted pendulum, is proposed. We investigated the stability of a linearized system. It was proven that the stabilizing control of the pendulum, based on the feedback principle, enables the stabilization of the system. We have identified the zones of stability and the amplitude–frequency characteristics. In the second part of this article, a generalization of the dynamic system for the case of the hysteresis friction in the mechanical joint is considered. The role of nonlinear effects within the design Preisach model and the phenomenological Bouc–Wen model is shown.

## 1. Introduction

To this date, improvements in the design and performance of energy harvesting systems are the subject of intense research. Modern advances in materials science, electronics, and control theory have reduced the size, reliability, and cost of such devices; nevertheless, achieving a performance comparable to traditional electric batteries continues to be an urgent scientific and technological challenge.

The process of energy saving, as it is known, consists of a redistribution of kinetic energy of an oscillating massive body into the form of electric energy. The non-linearity of the characteristics of the mechanical and/or electrical subsystems is of crucial significance. The importance of non-linear links in energy converters is emphasized in the works [1,2,3,4]. After all, linear systems have a sufficient coefficient of efficiency only under the condition of approximate equality of proper frequencies of mechanical and electrical subsystems, i.e., under the condition of closeness to the resonance mode of functioning [5,6]. Systematic changes in mechanical parameters are inevitably observed in industrial conditions, arising due to mechanical wear and ageing of materials and leading to degeneration of transfer characteristics. Let us also note the important influence of external noises, which can be correctly assessed only by probabilistic and statistical methods, for which we have to introduce probabilistic characteristics for individual system components [7].

Various mechanisms for maintaining resonance modes by means of vibrating oscillating systems with tunable characteristics were considered in the works [8,9,10,11,12,13,14]. However, the systems considered in these works require precision parameter tuning, which is not always feasible in real technical systems. In particular, much attention has been paid to the values of electric voltage [15], temperature conditions [16], and noise exposure [17,18].

In particular, a number of works are devoted to electric circuits that include piezoelectric materials, i.e., substances in which electric polarization arises under elastic deformation. They are crystalline substances without a center of symmetry and are characterized by a complex, non-linear relationship between the applied mechanical stress and the generated electric field. Among piezoelectrics there is the class of ferroelectric materials, which have a non-zero polarization in a certain temperature range, changing due to external excitation [19,20]. In fact, ferroelectrics are the material used to build microelectromechanical systems (MEMS), sound emitters, accelerometers, and precision microdisplacement sensors [16,21,22,23,24,25,26,27].

The control problem was considered in the articles [28,29,30]. The characteristics of chaotic dynamic modes arising from certain parameters of the system were investigated in [31,32,33,34].

Relatively small mechanical voltages lead to a linear response of the electrical characteristics of the ferroelectric material. On the other hand, in the practical issue of energy harvester design, as a rule, the values of external excitation have to exceed the linear response threshold. Thus, there is a hysteresis dependence of voltage, charge, and other electrical characteristics on the dynamic parameters of the mechanical subsystem [35,36].

A hysteresis loop occurs during consideration of the dependence of the polarization of a ferroelectric material sample on the applied electric field, whereas in an alternating field, the loop parameters depend considerably on the frequency of field change. Furthermore, a hysteresis relationship appears in the volt-farad characteristics of some ferroelectric films, i.e., the capacity depends on the voltage applied to the sample [37,38]. It is quite interesting that the samples of ferroelectric films sprayed on silicon plates of *p*- and *n*-type conductivity differ in the direction of loop bypass (clockwise and counter-clockwise, respectively). Hysteresis volt-farad characteristics are also demonstrated by metal-oxide-semiconductors (MOS) [39] and crystalline copolymer thin films) [40].

Dynamic modeling of systems with hysteresis is a complex mathematical problem attracting the attention of many researchers. Design models include the non-ideal relay, Preisach operator, and Ishlinsky model [41,42,43,44,45,46], and phenomenological models include the Bouc–Wen, Ivan, Duhem, etc. [47,48,49].

Among the structural models of hysteresis non-linearities, the Preisach operator [50,51,52] plays an important role. This model was originally formulated to describe the properties of ferromagnetic materials [53]; later its applicability to a wide range of phenomena was proved from various scientific and practical problems [54,55]. The Preisach model is based on a non-linear operator, which is a continuum system of relays connected in parallel. The output of the Preisach operator Γ[u(t)] (where u(t) is a continuous function of time) is a function x(t), whose value at each time moment is determined, as for all hysteresis converters, not only by the input u(t), but also by the background.

The Bouc–Wen model is one of the most commonly used phenomenological hysteresis models [48,56]. This model is based on the consideration of a one-dimensional mechanical system in which the return force, $\Phi_{BW}\left(x,t\right)$, applied to a material point is represented as the sum of two components:

(1) $$\Phi_{BW}\left(x,t\right)=\alpha kx\left(t\right)+\left(1-\alpha \right)Dkz\left(t\right),$$

where x(t) is the displacement of the body along the coordinate axis, αkx(t) is the elastic return force, *k* is the stiffness factor, (1−α)Dkz(t) is the hysteresis term, α is the relative stiffness, i.e., the ratio of the strain above the elastic threshold to the strain below the elasticity, and *D* is the plastic displacement strain. The auxiliary dimensionless function, z(t), included in the right side of Equation (1), satisfies a differential equation of first order:

(2) $$\dot{z}\left(t\right)=D^{-1}(A\dot{x}-\beta \dot{x}{\left|z\right|}^{n}-\gamma |\dot{x}{\left||z\right|}^{n-1}z),$$

which includes the time derivative, $\dot{x}\left(t\right)$, of the displacement on the right hand side. The mapping of $x\left(t\right)\to \Phi_{BW}\left(x,t\right)$ thus constructed is called the hysteresis model of Bouc–Wen [48].

Note that the integer parameter *n* determines the smoothness of the transition from elastic to plastic deformation. The parameters *A*, β, and γ are also dimensionless; they determine the shape of the hysteresis loop.

## 2. Energy Converter Model in Linear Approximation

As stated above, the most important parts of the energy converter are the mechanical oscillating system and the associated electromagnetic system, perceiving the oscillation energy. In this paper, it is proposed to choose the mechanical part of the energy converter in the form of an inverse pendulum.

**Figure 1:**
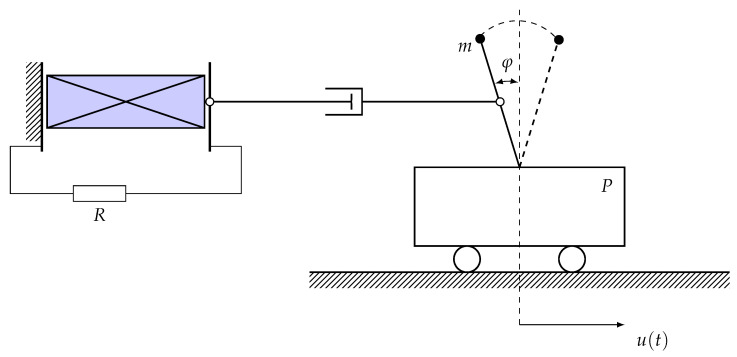
Schematic description of a mathematical pendulum connected to a piezoelectric generator.

The model of the investigated system consists of an inverted mathematical pendulum fixed on a light horizontal platform and mechanically connected to a ferroelectric condenser, which is included in a closed electrical circuit. The platform *P* can move horizontally. We denote the angle of deviation of the pendulum with respect to the vertical by φ(t), the coordinate of the platform by u(t) (see Figure 1), the length of the pendulum as *l*, and its mass as *m*. The connection between the mechanical and electrical subsystems contains a joint obeying the law of viscous friction with a dissipation factor *c*$\left[F_{\mathrm{fr}}=cv\left(t\right)=c\frac{d}{dt}\left(l\varphi /2+u\right))\right]$.

Piezoelectric material forming a condenser with capacitance *C* is included in an electric circuit with an external ohmic load of *R*. We denote the voltage at the load by V(t).

The dynamic system under consideration is described by a system of ordinary differential equations:

(3) $$\left\{\begin{array}{cc} \; & ml\ddot{\varphi }+c\left(l\dot{\varphi }/2+\dot{u}\right)-mg\sin\varphi +A\;V=-m\ddot{u},\\ \; & C\dot{V}+\frac{V}{R}=B\;\left(l\dot{\varphi }/2+\dot{u}\right). \end{array}\right.$$

The first of the equations in (3) is the equation of motion of weight *m* under the effect of mechanical and inertial forces, and the second is the balance of currents in an electric circuit. The dot above the symbol indicates the time derivative of *t*.

Assuming small deviations of the pendulum from the equilibrium position (sinφ∼φ), we obtain (where we have further introduced the notations $\gamma_{0}=c/\left(2m\right)$, $\omega_{0}^{2}=g/l$):

(4) $$\left\{\begin{array}{cc} \; & \ddot{\varphi }+\gamma_{0}\dot{\varphi }-\omega_{0}^{2}\varphi +\frac{A}{ml}V=-\frac{1}{l}\ddot{u}-2\frac{\gamma_{0}}{l}\dot{u},\\ \; & \dot{V}+\frac{1}{RC}V-\frac{Bl}{2C}\dot{\varphi }=\frac{B}{C}\dot{u}. \end{array}\right.$$

In this system, *A* and *B* are the coupling parameters of the mechanical and electrical subsystems, respectively, which are derived on the basis of the following considerations. The equation with respect to the unknown function φ(t) is the equation of motion of the exited oscillator (additionally to mechanical forces) by external force of nonmechanical nature ($F_{\mathrm{ext}}\left(t\right)=A\;V\left(t\right)$) and the inertial force $F_{\mathrm{iner}}\left(t\right)=-m\ddot{u}\left(t\right)$.

The elastic strain law (Hooke’s law) relates tension σ and the relative strain (for moderate absolute values of elastic strain):

(5) σ=Eε,

where *E* is the Young’s modulus, depending only on the piezoelectric material and its physical state (in the non-linear case $\sigma =E\varepsilon +E_{2}\varepsilon^{2}+O\left(\varepsilon^{2}\right)$). If there are no tangential stresses, the polarization of the piezoelectric specimen under tension or compression is determined by the expression:

(6) $$P_{x}=d_{11}\left(\tau_{x}-\tau_{y}\right),$$

where $\tau_{x}$ and $\tau_{y}$ are the mechanical tensions acting parallel to the axes Ox and Oy, respectively, and $d_{11}$ is the constant called the piezoelectric modulus. Express the value of the charge Q=CV(t) generated on the surfaces of the sample (taking *S* as the area of one surface and *C* as the electrical capacitance of the condenser formed by the piezoelectric plate):

(7) $$Q=p_{x}S,$$

(8) $$Q=d_{11}F.$$

There is a coupling between the elastic force acting from the piezoelectric on the weight *m* and the displacement δ(t)=lφ/2+u in the form of linear relation:

(9) $$F=\frac{d_{11}}{C}V=A\;V,$$

where *A* is a constant which depends only on the material and its dielectric thermodynamic properties. In other words, the voltage depends linearly on the displacement by the formula $V\left(\delta \right)=\frac{ES}{AL}\delta \left(t\right)$, where *L* is the length of the piezoelectric plate (as Hooke’s law can be represented as F/S=E×(δ/L)).

To take advantage of the polarization charges occurring on the opposite surfaces of the quartz plate during its deformation, the surfaces are provided with metallic covers. Charges equal and opposite in sign to polarizing charges are induced on such covers, and electric current is generated in the external wires connecting the covers.

In an electric circuit consisting of an external current source (due to polarization of the dielectric plates) and connected condenser *C* and resistor *R* in parallel (which acts as an active load), Ohm’s law applies:

(10) $$C\dot{V}+\frac{V}{R}=B\dot{\delta },$$

where $B=\frac{CES}{AL}=\frac{C^{2}ES}{d_{11}L}$ is the constant.

Note that the literature on energy storage models describes electrical subsystems built on the same principles, but satisfying other differential equations:

For example, in ref. [4]:

(11) $$\tilde{B}\ddot{x}+C\ddot{V}+\frac{\dot{V}}{R}+\frac{V}{L}=0,$$

and in refs. [57,58]:

(12) $$R\dot{Q}-\frac{d(x)}{C}x+\frac{Q}{C}=0,$$

where d(x) is the coupling coefficient between the mechanical and electrical subsystems.

Among the many options for mechanical pendulum-based oscillation systems proposed for use in energy harvesters we note the inverted pendulum beam [59], the inverted pendulum with amplitude limiters [60], and the inverted cantilever beam with a tip mass [61].

In work [59] it was proposed to construct an energy storage device in the form of an inverted piezoelectric beam and a pendulum. A layer of piezoelectric material was attached to the base of the beam, which is subject to external disturbance in the horizontal direction. Due to the instability of the inverted beam, even weak excitation leads to oscillations with significant amplitude.Additionally, the existence of two oscillation modes, between which there is a mechanical connection, allows the transmission of high-frequency excitation energy to a low-frequency mode, which generates electrical power.

The model used in ref. [60] was based on a vertical solid flexible beam with a massive tip. Instead of the standard clamp, a joint with an additional gap has been introduced.Due to the presence of contacts with limiters and movement around the hinge point, the pendulum was a system with two modes of oscillation: rotation and bending. Calculations showed that the potential energy of the system was characterized by two potential wells, and the responses are subharmonic solutions with multiples of odd frequencies. The authors demonstrated that this mechanical system could be used as a resonator in a broadband energy collector.

A similar device was discussed earlier in ref. [61], where the linear case was analyzed in detail. It was shown that if the beam has a strong bend, then the system shows nonlinear characteristics, including a chaotic regime.

The inverted pendulum is also a key element in the storage of biomechanical energy in a backpack device [62]. It generates energy due to medial-lateral oscillations of carried weight. In general, the concept of an inverted pendulum arises naturally when considering human walking modelling to harvest electrical energy [63].

### 2.1. Converting the System to a Dimensionless Form

Let us switch from variables *t* and *V* to dimensionless variables by the following rule:

(13) $$t=t_{c}\cdot \tau ,\;V=V_{c}\cdot v,$$

where the characteristic values of time, $t_{c}$, and voltage, $V_{c}$, will be determined below. (The deflection angle of the pendulum, φ(t), is a dimensionless value.)

The system (3) takes the following form:

(14) $$\left\{\begin{array}{cc} \; & \frac{1}{t_{c}^{2}}\ddot{\varphi }+\frac{\gamma_{0}}{t_{c}}\dot{\varphi }-\omega_{0}^{2}\varphi +\frac{A}{ml}V_{c}v=-\frac{1}{lt_{c}^{2}}\ddot{u}\left(t_{c}\tau \right)-2\frac{\gamma_{0}}{lt_{c}}\dot{u}\left(t_{c}\tau \right),\\ \; & \frac{V_{c}}{t_{c}}\dot{v}+\frac{1}{RC}V_{c}v-\frac{Bl}{2C}\frac{1}{t_{c}}\dot{\varphi }=\frac{B}{Ct_{c}}\dot{u}\left(t_{c}\tau \right). \end{array}\right.$$

Hereinafter, the point above the symbol denotes the derivative of the dimensionless time τ (two points denotes the second derivative of τ). Let us perform algebraic transformations:

(15) $$\left\{\begin{array}{cc} \; & \ddot{\varphi }+\gamma_{0}t_{c}\dot{\varphi }-\omega_{0}^{2}t_{c}^{2}\varphi +\frac{A}{ml}V_{c}t_{c}^{2}v=-\frac{1}{l}\ddot{u}\left(t_{c}\tau \right)-2\frac{\gamma_{0}t_{c}}{l}\dot{u}\left(t_{c}\tau \right),\\ \; & \dot{v}+\frac{1}{RC}t_{c}v-\frac{Bl}{2C}\frac{1}{V_{c}}\dot{\varphi }=\frac{B}{CV_{c}}\dot{u}\left(t_{c}\tau \right). \end{array}\right.$$

Let the function defining the motion of the platform *P* be $\tilde{u}\left(t\right)=u\left(t_{c}\tau \right)$, and the values of dimensional coefficients be $t_{c}=RC$ and $V_{c}=\frac{ml}{A{\left(RC\right)}^{2}}$. Then, we get the basic system in a dimensionless form:

(16) $$\left\{\begin{array}{cc} \; & \ddot{\varphi }+\gamma_{0}\left(RC\right)\dot{\varphi }-\omega_{0}^{2}{\left(RC\right)}^{2}\varphi +v=-\frac{1}{l}\ddot{\tilde{u}}\left(\tau \right)-2\frac{\gamma_{0}\left(RC\right)}{l}\dot{\tilde{u}}\left(\tau \right),\\ \; & \dot{v}+v-\frac{AB}{2m}R^{2}C\dot{\varphi }=\frac{AB}{ml}R^{2}C\dot{\tilde{u}}\left(\tau \right). \end{array}\right.$$

To simplify and clarify further calculations, we introduce notations for dimensionless values of damping coefficient, characteristic frequency of the pendulum, and external influence as $\gamma =\gamma_{0}\cdot \left(RC\right)$, $\omega =\omega_{0}\cdot \left(RC\right)$, and $w=\tilde{u}\left(\tau \right)/l$, respectively. It is also convenient to denote the coupling coefficient of the electrical and mechanical subsystems by $\sigma =\frac{AB}{2m}R^{2}C$. Conclusively,

(17) $$\left\{\begin{array}{cc} \; & \ddot{\varphi }+\gamma \dot{\varphi }-\omega^{2}\varphi +v=-\ddot{w}\left(\tau \right)-2\gamma \dot{w}\left(\tau \right),\\ \; & \dot{v}+v-\sigma \dot{\varphi }=2\sigma \dot{w}\left(\tau \right), \end{array}\right.$$

or, in a more convenient matrix form:

(18) $$\frac{d}{d\tau }\left[\begin{array}{c} \varphi \\ \psi \\ v \end{array}\right]=\left[\begin{array}{ccc} 0 & 1 & 0\\ \omega^{2} & -\gamma & -1\\ 0 & \sigma & -1 \end{array}\right]\left[\begin{array}{c} \varphi \\ \psi \\ v \end{array}\right]+\left[\begin{array}{c} 0\\ -\ddot{w}-2\gamma \dot{w}\\ 2\sigma \dot{w} \end{array}\right],$$

where $\psi \left(\tau \right)=\dot{\varphi }\left(\tau \right)$ is the angular velocity of the pendulum. Note that according to the physical formulation of the problem, in inequalities ω>0 and γ⩾0 are satisfied.

### 2.2. Investigation of the Stability of a Linearized System

Investigate the stability of the solutions of the system (18). To do this, we write out the characteristic equation for (18) (we consider the autonomous case):

(19) $$\left|\begin{array}{ccc} -\lambda & 1 & 0\\ \omega^{2} & -\gamma -\lambda & -1\\ 0 & \sigma & -1-\lambda \end{array}\right|=0.$$

When converted to a third-degree algebraic equation with constant coefficients it takes the form:

(20) $$\lambda^{3}+\left(\gamma +1\right)\lambda^{2}+\left(\gamma +\sigma -\omega^{2}\right)\lambda -\omega^{2}=0.$$

As is known, a necessary and sufficient condition for stability of a system of differential equations is the positivity of all three main diagonal minors $\Delta_{1}$, $\Delta_{2}$, and $\Delta_{3}$ of the Hurwitz determinant

(21) $$\Gamma =\left|\begin{array}{ccc} a_{1} & a_{3} & 0\\ a_{0} & a_{2} & 0\\ 0 & a_{1} & a_{3} \end{array}\right|,$$

where $a_{i}$, i∈{0,1,2,3} are coefficients in (20); moreover, $a_{0}=1$ (see, for example, ref. [64]). Since $a_{3}=-\omega^{2}<0$, the conditions in Hurwitz criterion $\Delta_{1}>0$, $\Delta_{2}>0$, and $\Delta_{3}>0$,

(22) $$\left\{\begin{array}{c} a_{1}>0,\\ a_{1}a_{2}-a_{0}a_{3}>0,\\ a_{3}\left(a_{1}a_{2}-a_{0}a_{3}\right)>0, \end{array}\right.$$

as it can be easily seen, are incompatible for any admissible values of the parameters ω, γ, and σ. Herewith, system (18) is unstable.Nevertheless, the introduction of a control with external force w(τ) makes it possible in some cases to bring the dynamical system into a stable oscillation mode. In the next section, we will consider at what values of the parameters of the problem this is possible.

### 2.3. Amplitude Control of the Pendulum Oscillation

In this section, we will show that the introduction of a control by external force w(τ) makes it possible to stabilize the motion of the pendulum and provides an opportunity to control amplitude φ(τ) of its oscillations.

In this section, we use the representation of the values varying according to the harmonic law in the form of the real part of the exponential function of the imaginary argument ∼exp(iΩτ). It is known, for linear systems of algebraic or differential equations, this approach is correct and allows to perform intermediate calculations in a more compact and clear form.

Let us write the system of Equation (18) under control conditions in the form $\dot{w}\left(t\right)=\alpha \varphi \left(t\right)+d\;e^{i\Omega t}$, where d∈C:

(23) $$\frac{d}{d\tau }\left[\begin{array}{c} \varphi \\ \psi \\ v \end{array}\right]=\left[\begin{array}{ccc} 0 & 1 & 0\\ \omega^{2} & -\gamma & -1\\ 0 & \sigma & -1 \end{array}\right]\left[\begin{array}{c} \varphi \\ \psi \\ v \end{array}\right]+\left[\begin{array}{c} 0\\ -\alpha \dot{\varphi }-2\gamma \alpha \varphi -e^{i\Omega \tau }\left(i\Omega +2\gamma \right)d\\ 2\sigma \alpha \dot{\varphi }+2\sigma i\Omega e^{i\Omega \tau }d \end{array}\right],$$

or

(24) $$\frac{d}{d\tau }\left[\begin{array}{c} \varphi \\ \psi \\ v \end{array}\right]=\left[\begin{array}{ccc} 0 & 1 & 0\\ \omega^{2}-2\gamma \alpha & -(\gamma +\alpha ) & -1\\ 2\sigma \alpha & \sigma & -1 \end{array}\right]\left[\begin{array}{c} \varphi \\ \psi \\ v \end{array}\right]+\left[\begin{array}{c} 0\\ -\left(i\Omega +2\gamma \right)e^{i\Omega \tau }\\ 2\sigma i\Omega e^{i\Omega \tau } \end{array}\right]d.$$

As can be seen from (24), the coefficients of the matrix have changed as a result of the control excitation. Check the conditions of the Hurwitz criterion in this case. Write out the characteristic equation

(25) $$\lambda^{3}+\left(1+\gamma +\alpha \right)\lambda^{2}+\left[\gamma +\sigma -\omega^{2}+\left(1+2\gamma \right)\alpha \right]\lambda +\left[-\omega^{2}+2\left(\gamma +\sigma \right)\alpha \right]=0,$$

and the Hurwitz matrix

(26) $$\Gamma \left(\alpha \right)=\left[\begin{array}{ccc} 1+\gamma +\alpha & -\omega^{2}+2\left(\gamma +\sigma \right)\alpha & 0\\ 1 & \gamma +\sigma -\omega^{2}+\left(1+2\gamma \right)\alpha & 0\\ 0 & 1+\gamma +\alpha & -\omega^{2}+2\left(\gamma +\sigma \right)\alpha \end{array}\right],$$

which leads to the following conditions:

(27) $$\left\{\begin{array}{c} 1+\gamma +\alpha >0,\\ \left(1+2\gamma \right)\alpha^{2}+\left(1+2\gamma +2\gamma^{2}-\sigma -\omega^{2}\right)\alpha +\left(1+\gamma \right)\left(\gamma +\sigma \right)-\gamma \omega^{2}>0,\\ -\omega^{2}+2\left(\gamma +\sigma \right)\alpha >0. \end{array}\right.$$

Therefore, system (24) is stable if and only if its parameters satisfy conditions (27). Its solution with respect to control parameter α has a simple form:

(28) $$\left\{\begin{array}{c} \alpha >\frac{\omega^{2}}{2(\gamma +\sigma )},\;\mathrm{if}\;D>0,\\ \alpha >\max\left(\frac{\omega^{2}}{2(\gamma +\sigma )},\alpha_{b}\right),\;\mathrm{if}\;D⩽0, \end{array}\right.$$

where $\alpha_{b}$ is the larger of two roots of (27) and D is its discriminant:

(29) $$D={\left(1+2\gamma +2\gamma^{2}-\sigma -\omega^{2}\right)}^{2}-4\left(1+2\gamma \right)\left(\gamma +\gamma^{2}+\sigma +\gamma \sigma -\gamma \omega^{2}\right).$$

Write down the solution to system (24), represented as a sum of the general solution of the homogeneous system and the partial solution of the inhomogeneous one:

(30) φψv=Re(C1eλ1τf→1+C2eλ2τf→2+C3eλ3τf→3+AφAψAveiΩτ).

In (30), $\lambda_{1}$, $\lambda_{2}$, and $\lambda_{3}$ are the eigenvalues of matrix (24) and ${\vec{f}}_{1}$, ${\vec{f}}_{2}$, and ${\vec{f}}_{3}$ are corresponding eigenvectors.

In a steady-state, i.e., at sufficiently large values of the time, τ≫max(1/|Reλ1|, 1/|Reλ2|,1/|Reλ3|),

(31) φψv=ReAφAψAveiΩτ.

As can be seen from (31), the solution in the steady-state condition contains terms varying according to the harmonic law $∼e^{i\Omega \tau }$. Their amplitudes $A_{\varphi }$, $A_{\psi }$, and $A_{v}$, as it can be easily shown by direct substitution of $\{\varphi \left(\tau \right)=A_{\varphi }e^{i\Omega \tau }$, $\psi \left(\tau \right)=A_{\psi }e^{i\Omega \tau }$, $v\left(\tau \right)=A_{v}e^{i\Omega \tau }\}$ in (24), satisfy the system of algebraic equations:

(32) $$\left[\begin{array}{ccc} -i\Omega & 1 & 0\\ \omega^{2}-2\gamma \alpha & -(\gamma +\alpha +i\Omega ) & -1\\ 2\sigma \alpha & \sigma & -1-i\Omega \end{array}\right]\left[\begin{array}{c} \varphi \\ \psi \\ v \end{array}\right]+\left[\begin{array}{c} 0\\ -(i\Omega +2\gamma )\\ 2\sigma i\Omega \end{array}\right]d=0.$$

Therefore, for the amplitude of oscillations of the pendulum as a function of the amplitude of the harmonic term with frequency Ω in the control $\dot{w}\left(\tau \right)$ we get the expression:

(33) $$A_{\varphi }=\frac{1}{F(\Omega )}\left[2\gamma +i\left(1+2\gamma +2\sigma \right)\Omega -\Omega^{2}\right]d,$$

where $F\left(\Omega \right)=\omega^{2}-2\alpha \left(\gamma +\sigma \right)-i\Omega \left[\left(1+2\gamma \right)\alpha +\gamma +\sigma -\omega^{2}\right]+\Omega^{2}\left(\alpha +\gamma +1\right)+i\Omega^{3}$ is the determinant of (32) (note that this determinant can also be obtained from the right side of (25) by performing the formal substitution λ→iΩ). Steady-state oscillations are possible under condition F(Ω)≠0.

The amplitudes of oscillations of the angular velocity of the pendulum and the voltage are calculated in the same way:

(34) $$A_{\psi }=\frac{i\Omega }{F(\Omega )}\left[2\gamma +i\left(1+2\gamma +2\sigma \right)\Omega -\Omega^{2}\right]d,$$

(35) $$A_{v}=\frac{\sigma }{F(\Omega )}\left[4\alpha \gamma +2i\left(\alpha +\gamma -2\alpha \gamma +\omega^{2}\right)\Omega +\left(2\alpha +2\gamma -1\right)\Omega^{2}+2i\Omega^{3}\right]d.$$

**Figure 2:**
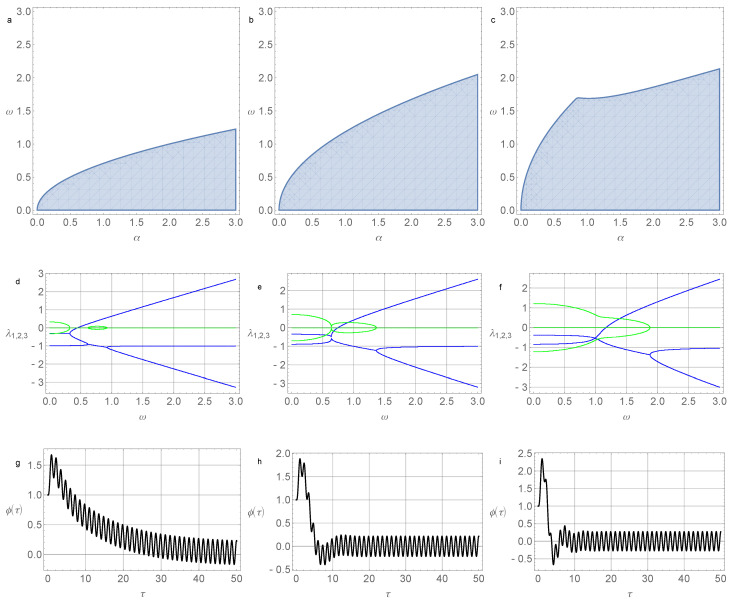
(**a**) System stability area (24) in the (α,ω) plane for parameter values σ=0.05 and γ=0.2; (**b**) system stability area (24) for parameter values σ=0.5 and γ=0.2; (**c**) system stability area (24) for parameter values σ=1.5 and γ=0.2; (**d**) real (thick blue lines) and imaginary parts (thin green lines) of the roots of the characteristic Equation (25) for σ=0.05, γ=0.2, α=0.4; (**e**) real (thick blue lines) and imaginary parts (thin green lines) of the roots of the characteristic Equation (25) for σ=0.5, γ=0.2, and α=0.4; (**f**) real (thick blue lines) and imaginary parts (thin green lines) of the roots of the characteristic Equation (25) for σ=1.5, γ=0.2, and α=0.4; (**g**) the angle of pendulum deviation depending on the dimensionless time for σ=0.05, γ=0.2, α=0.4, ω=0.4, d=1.0, and Ω=5.0; (**h**) the angle of pendulum deviation depending on the dimensionless time for σ=0.5, γ=0.2, α=0.4, ω=0.4, d=1.0, and Ω=5.0; (**i**) the angle of pendulum deviation depending on the dimensionless time for σ=1.5, γ=0.2, α=0.4, ω=0.4, d=1.0, and Ω=5.0. Initial conditions in (**g**,**h**,**i**) are φ(0)=1.0, ψ(0)=0.0, and v(0)=1.0.

**Figure 3:**
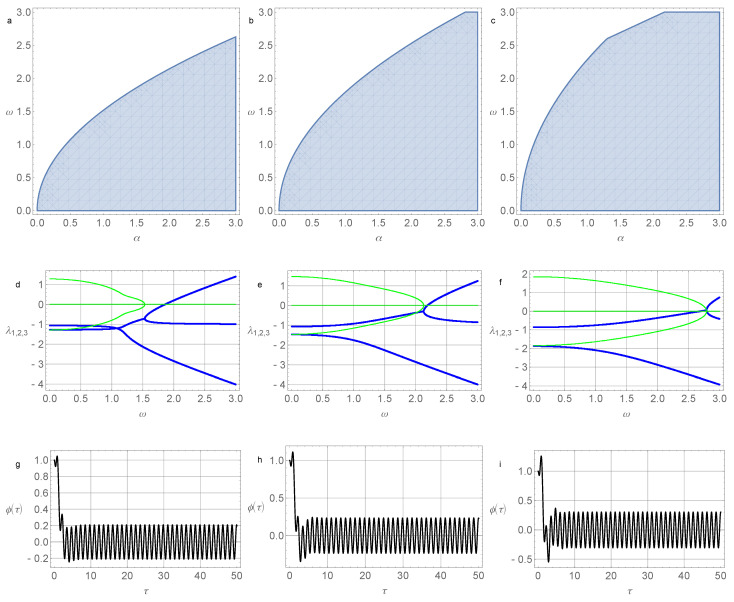
(**a**) System stability area (24) in the (α,ω) plane for parameter values σ=0.05 and γ=1.1; (**b**) system stability area (24) for parameter values σ=0.5 and γ=1.1; (**c**) system stability area (24) for parameter values σ=1.5 and γ=1.1; (**d**) real (thick blue lines) and imaginary parts (thin green lines) of the roots of the characteristic Equation (25) for σ=0.05, γ=1.1, and α=1.5; (**e**) real (thick blue lines) and imaginary parts (thin green lines) of the roots of the characteristic Equation (25) for σ=0.5, γ=1.1, and α=1.5; (**f**) real (thick blue lines) and imaginary parts (thin green lines) of the roots of the characteristic Equation (25) for σ=1.5, γ=1.1, and α=1.5; (**g**) the angle of pendulum deviation depending on the dimensionless time for σ=0.05, γ=1.1, α=1.5, ω=1.2, d=1.0, and Ω=5.0; (**h**) the angle of pendulum deviation depending on the dimensionless time for σ=0.5, γ=1.1, α=1.5, ω=1.2, d=1.0, and Ω=5.0; (**i**) the angle of pendulum deviation depending on the dimensionless time for σ=1.5, γ=1.1, α=1.5, ω=1.2, d=1.0, and Ω=5.0. Initial conditions in (**g**,**h**,**i**) are φ(0)=1.0, ψ(0)=0.0, and v(0)=1.0.

The results of numerical simulations of the stability zones, roots of the characteristic equation, and solutions to the system at various parameter values are shown in Figure 2 and Figure 3.

**Figure 4:**
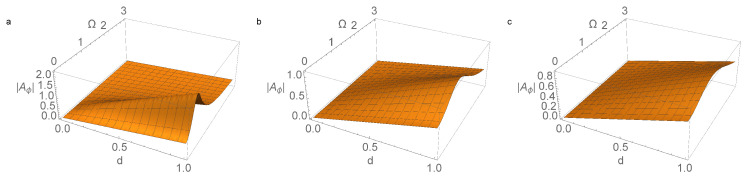
The amplitude of the forced oscillations of the pendulum $|A_{\varphi }\left(d,\Omega \right)|$ according to (33) for values of the parameter γ, characterizing friction: (**a**) γ=0.2; (**b**) γ=1.1; and (**c**) γ=1.9. The calculation was performed for σ=0.5, α=1.5, and ω=1.2.

**Figure 5:**
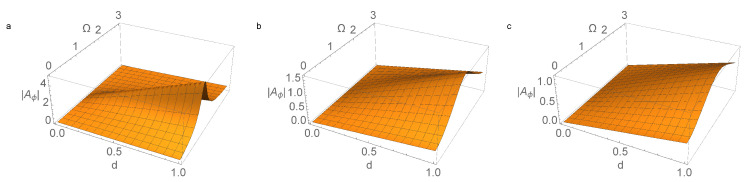
The amplitude of the forced oscillations of the pendulum $|A_{\varphi }\left(d,\Omega \right)|$ according to (33) for values of the parameter γ, characterizing friction: (**a**) γ=0.2; (**b**) γ=1.1; and (**c**) γ=1.9. The calculation was performed for σ=1.5, α=1.5, and ω=1.2.

Dependencies of the amplitude $|A_{\varphi }|$ of the forced oscillations in the parameter space {(d,Ω)} are shown in Figure 4 and Figure 5. These figures show the maximum amplitude decreasing with increasing γ, as well as the shift in the resonance region towards higher frequencies of Ω in this case. Due to the linearity of system (32), the value of $|A_{\varphi }|$ is proportional to the parameter *d* in the control function $\dot{w}\left(t\right)$.

## 3. Hysteresis Dependencies in the Energy Harvester Model

**Figure 6:**
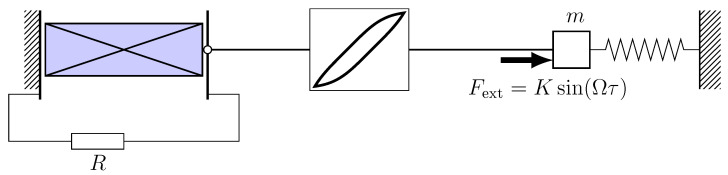
Energy harvester, mechanically connected to the classical oscillator by means of a hysteresis chain.

In the practical implementation of the design of energy harvesting devices, as a rule, there are hysteresis dependencies of various kinds, both in the mechanical subsystem and of an electromagnetic nature. In this section, we investigate an electromechanical energy harvesting system with hysteresis damping, in other words, it is assumed that the friction in the mechanical subsystem obeys the hysteresis law (see Figure 6). Below, we will look at two approaches to the formalization of hysteresis friction.

The first approach is based on the design model of the hysteresis operator (a Preisach operator), while the second one is described within the phenomenological Bouc–Wen model.

### 3.1. System with Hysteresis Friction within the Preisach Model

In Figure 6, a model of an energy harvester based on a classical oscillator *m*, connected to the electrical subsystem by means of a hysteresis damping link, is schematically presented. The external excitation was determined by periodic force $F_{\mathrm{ext}}=K\sin\left(\Omega \tau \right)$ applied to *m*.

In this section, we introduce a hysteresis operator using the approach developed in a book by M. A. Krasnoselskii and A. V. Pokrovskii. Within this approach, the hysteresis operator is interpreted as an operator defined on the space of continuous functions which dynamics are described by the relations: “input–state” and “state–output”.

Denote the hysteresis converter corresponding to the non-ideal relay with threshold numbers α and β by $R[\alpha ,\beta ,x_{0},t_{0}]$, where $x_{0}\in \left\{0,1\right\}$ is the initial state of the converter and $t_{0}$ is the initial time instant. The space of states of the non-ideal relay is a two-element set {0,1}. The input of the system is continuous at $t⩾t_{0}$ function u(t). The output is a a step function, x(t), defined by the relation:

(36) $$x\left(t\right)=R\left[\alpha ,\beta ,x_{0},t_{0}\right]u\left(t\right).$$

Note that initial state $x_{0}$ must satisfy the condition:

(37) x0=−0,ifu(0)⩽α,−1,ifu(0)⩾β.

In the case where inequalities α⩽u(0)⩽β are satisfied, value $x_{0}$ can achieve any value from set {0,1}. The output values of x(t) with continuous input u(t) for $t\in (t_{0},\infty )$ at each t=τ are determined following to the rule:

(38) $$R\left[\alpha ,\beta ,x_{0},t_{0}\right]u\left(\tau \right)=\left\{\begin{array}{c} \;\;x_{0},\;\mathrm{if}\;\forall t\in \left[t_{0},\tau \right]:\;\;\left[\alpha <u\left(t\right)<\beta \right],\\ \;\;1,\;\mathrm{if}\;\exists t^{\prime }\in \left[t_{0},\tau \right):\left[u\left(t^{\prime }\right)⩾\beta \right]\wedge \left\{\forall t\in \left[t^{\prime },\tau \right]:\;\left[u\left(t\right)>\alpha \right]\right\},\\ \;\;0,\;\mathrm{if}\;\exists t^{\prime }\in \left[t_{0},\tau \right):\left[u\left(t^{\prime }\right)⩽\alpha \right]\wedge \left\{\forall t\in \left[t^{\prime },\tau \right]:\;\left[u\left(t\right)<\beta \right]\right\}. \end{array}\right.$$

Let us say that the relay is on if the output is equal to one, and that the relay is off otherwise.

**Figure 7:**
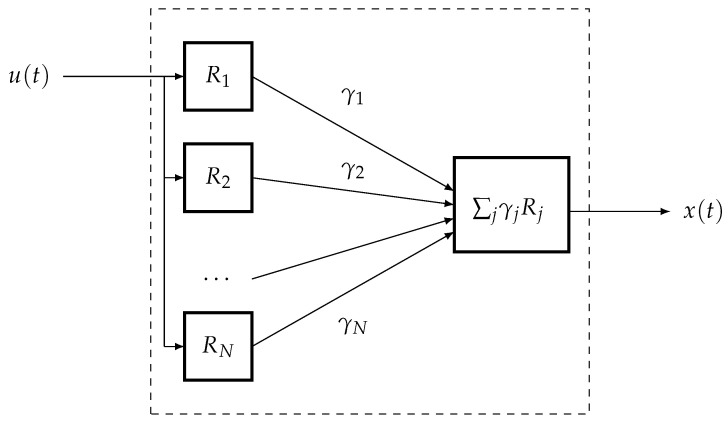
The parallel connection of *N* relay $R_{j}\left[\alpha ,\beta ,x_{0},t_{0}\right]$, taken with weights $\gamma_{j}>0$, where j=1,⋯,N.

The Preisach operator is a continuum analogue of a family of non-ideal relays connected in parallel (a block diagram of such a converter is shown in Figure 7). 

The state space of this operator consists of pairs {u(t),z(α,β,t)}, where u(t) is the input value at time instant *t* and z(α,β,t) is the characteristic function of the subset half-plane α<β, taking values 0,1. The input-output relationships of the Preisach operator are defined by the relations: input–state

(39) $$z\left(\alpha ,\beta ,t\right)=R\left[\alpha ,\beta ,z_{0},t_{0}\right]u\left(t\right),$$

where $z_{0}=z\left(\alpha ,\beta ,t_{0}\right)$, and state–output

(40) $$\Gamma \left[z\left(\alpha ,\beta ,t\right),u\left(t_{0}\right),t_{0}\right]u\left(t\right)=\underset{\alpha <\beta }{\;\int \int \;}z\left(\alpha ,\beta ,t\right)d\alpha d\beta .$$

The specified operator is widely used to formalize various hysteresis dependencies, its properties as well as its various applications can be found, for example, in [65].

Assume that the support of the measure of the Preisach operator is bounded, and therefore the space of states consists of characteristic functions, the support of which is contained in bounded sets.

**Figure 8:**
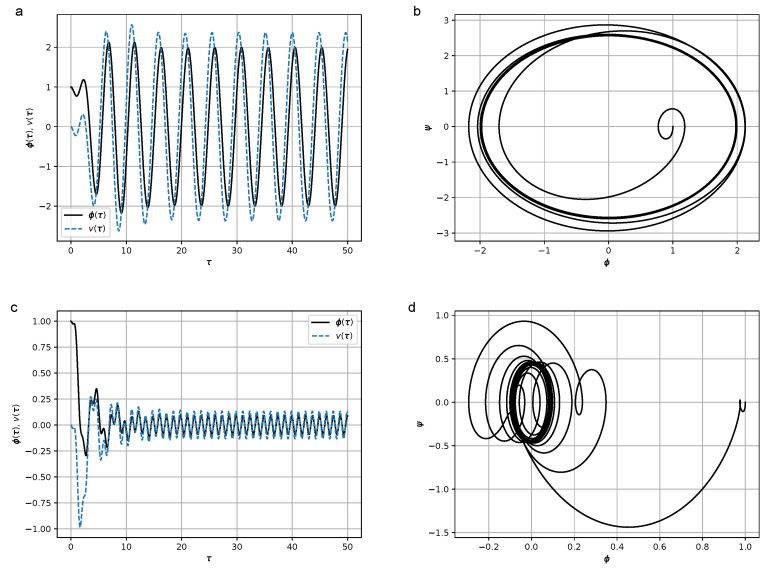
(**a**) Dependence of the declination angle of the pendulum, φ(τ), and the stress, v(τ), from the dimensionless time in the absence of the hysteresis term at Ω=1.3 and H=0; (**b**) phase portrait of the oscillations of the pendulum ψ(φ) at Ω=1.3 and H=0; (**c**) dependence of the declination angle of the pendulum, φ(τ), and the stress, v(τ), in the absence of the hysteresis term at Ω=5.0 and H=0; (**d**) phase portrait of the oscillations of the pendulum ψ(φ) at Ω=5.0 and H=0. Parameter values were σ=1.5, ω=1.2, and K=2.0. Initial conditions were φ(0)=1, ψ(0)=0, and σ(0)=0.

**Figure 9:**
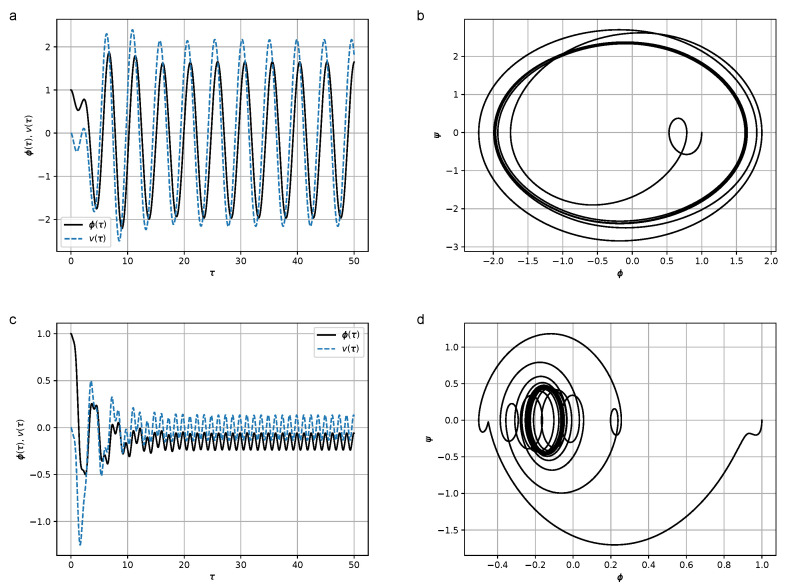
(**a**) Dependence of the declination angle of the pendulum, φ(τ), and the stress, v(τ), from the dimensionless time at Ω=1.3 and H=0.5; (**b**) Phase portrait of the oscillations of the pendulum ψ(φ) at Ω=1.3, H=0.5; (**c**) Dependence of the declination angle of the pendulum, φ(τ), and the stress, v(τ), from the dimensionless time at Ω=5.0 and H=0.5; (**d**) Phase portrait of the oscillations of the pendulum ψ(φ) at Ω=5.0, and H=0.5. Parameter values were σ=1.5, ω=1.2, and K=2.0. Initial conditions were φ(0)=1, ψ(0)=0, and σ(0)=0.

**Figure 10:**
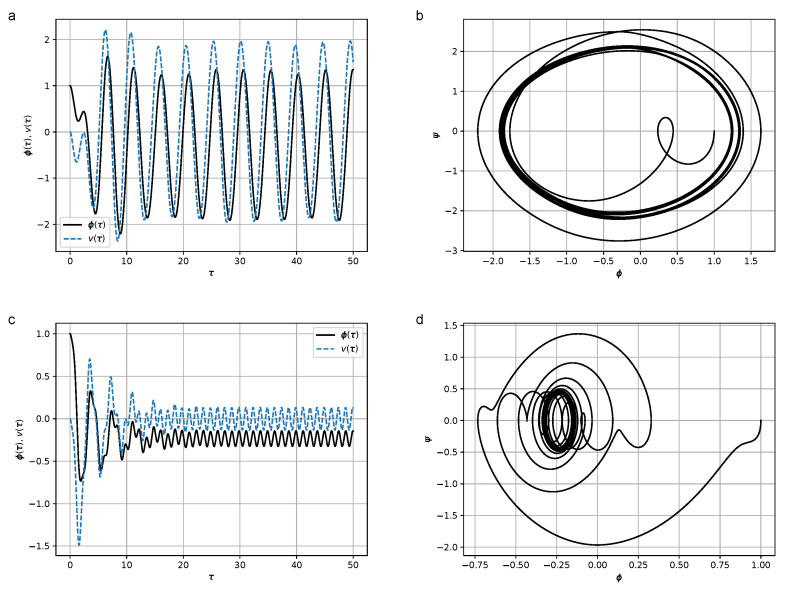
(**a**) Dependence of the declination angle of the pendulum, φ(τ), and the stress, v(τ), from the dimensionless time at Ω=1.3 and H=1.0; (**b**) Phase portrait of the oscillations of the pendulum ψ(φ) at Ω=1.3 and H=1.0; (**c**) Dependence of the declination angle of the pendulum, φ(τ), and the stress, v(τ), from the dimensionless time at Ω=5.0 and H=1.0; (**d**) Phase portrait of the oscillations of the pendulum ψ(φ) at Ω=5.0 and H=1.0. Parameter values qwew σ=1.5, ω=1.2, ans K=2.0. Initial conditions were φ(0)=1, ψ(0)=0, and σ(0)=0.

**Figure 11:**
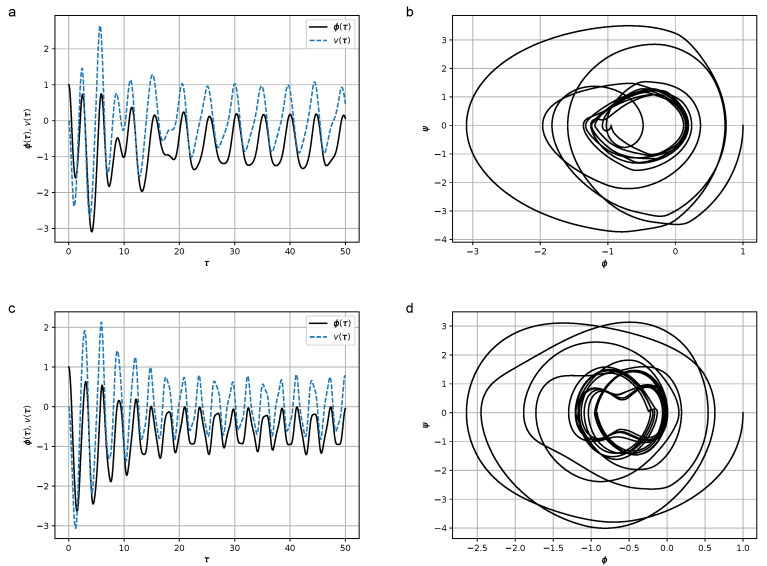
(**a**) Dependence of the declination angle of the pendulum, φ(τ), and the stress, v(τ), from the dimensionless time at Ω=1.3 and H=8.0; (**b**) Phase portrait of the oscillations of the pendulum ψ(φ) at Ω=1.3 and H=8.0; (**c**) Dependence of the declination angle of the pendulum, φ(τ), and the stress, v(τ), from the dimensionless time at Ω=5.0 and H=8.0; (**d**) Phase portrait of the oscillations of the pendulum ψ(φ) at Ω=5.0 and H=8.0. Parameter values were σ=1.5, ω=1.2, and K=2.0. Initial conditions were φ(0)=1, ψ(0)=0, and σ(0)=0.

The results of the numerical simulations of the system with hysteresis friction within the Preisach model

(41) $$\left\{\begin{array}{cc} \; & \ddot{\varphi }+H\Gamma \varphi \left(\tau \right)+\omega^{2}\varphi +v=K\sin\left(\Omega \tau \right),\\ \; & \dot{v}+v-\sigma \dot{\varphi }=0, \end{array}\right.$$

where *H* is the coefficient characterizing the effect of hysteresis friction, are shown in Figure 6. The results of numerical simulations at values H∈{0,0.5,1.0,8.0} are shown in Figure 8, Figure 9, Figure 10 and Figure 11. In the calculations, set [−1,1]×[−1,1] was chosen as the support of the Preisach operator measure, where the number of elementary hysterons was 20,100.

Figures show that in the frequency region Ω≫ω, the amplitude of oscillations is lower than in the resonant case Ω≃ω. At relatively high values of *H* (H=8) the duration of the transition process leading to steady-state oscillations increases.

**Figure 12:**
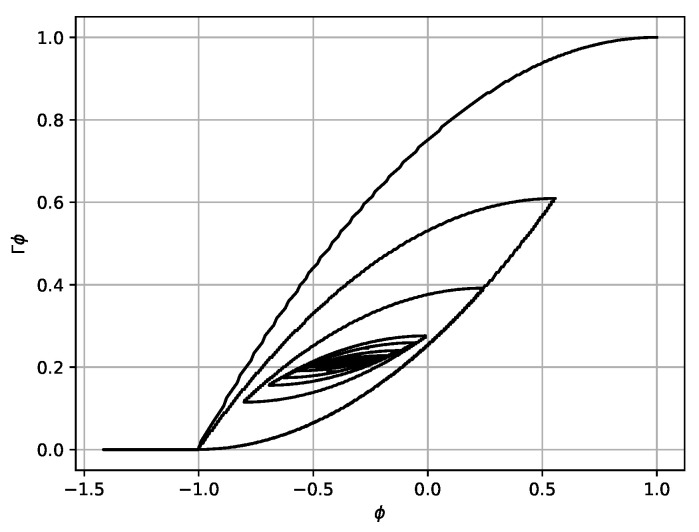
The dependence of Γφ on φ at parameter values K=2.0, ω=1.2, Ω=5.0, σ=1.5, and H=2.5. Initial conditions were φ(0)=1, ψ(0)=0, and σ(0)=0.

Figure 12 shows the hysteresis curve in coordinates (φ,Γφ). As can be seen, after several oscillation cycles the established mode is reached. The values of transmitted power and the electrical power are presented in Table 1.

### 3.2. Bouc–Wen Model

Within the Bouc–Wen model the dynamics of the energy harvester with hysteresis friction will be described by (42):

(42) $$\left\{\begin{array}{c} \ddot{\varphi }+Hz+\omega^{2}\varphi +v=K\sin\left(\Omega \tau \right),\\ \dot{v}+v-\sigma \dot{\varphi }=0,\\ \dot{z}=A\dot{\varphi }-\beta \dot{\varphi }{\left|z\right|}^{n}-\gamma |\dot{\varphi }{\left||z\right|}^{n-1}z. \end{array}\right.$$

**Figure 13:**
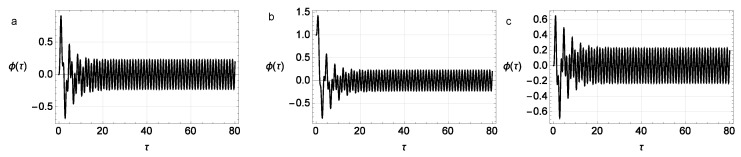
The dependence of the deflection angle of the pendulum, φ(τ), on various initial conditions at ω=1.2, H=0.2, K=5, Ω=5, and σ=1.5. Parameters of Bouc–Wen model: A=1, β=0.1, γ=0.9, and n=2. Initial conditions are different in different panels of the figure: (**a**) φ(0)=0 and $\dot{\varphi }\left(0\right)=v\left(0\right)=z\left(0\right)=0$; (**b**) φ(0)=1 and $\dot{\varphi }\left(0\right)=0$, z(0)=0; (**c**) $\varphi \left(0\right)=\dot{\varphi }\left(0\right)=0$, v(0)=1, and z(0)=0.

**Figure 14:**
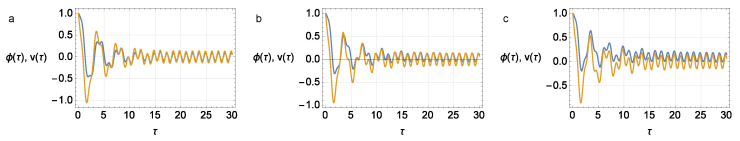
The dependence of the deflection angle of the pendulum, φ(τ), and the stress, v(τ), on the dimensionless time at different values of the coefficient at the hysteresis term; ω=1.2, K=2, Ω=5, and σ=1.5. Parameters of Bouc–Wen model: A=1, β=0.1, γ=0.9, and n=3 and initial conditions φ(0)=1, v(0)=1, and (**a**) H=0.0, (**b**) H=0.5, or (**c**) H=1.

The numerical results for (42) are shown in Figure 13 and Figure 14.

**Figure 15:**
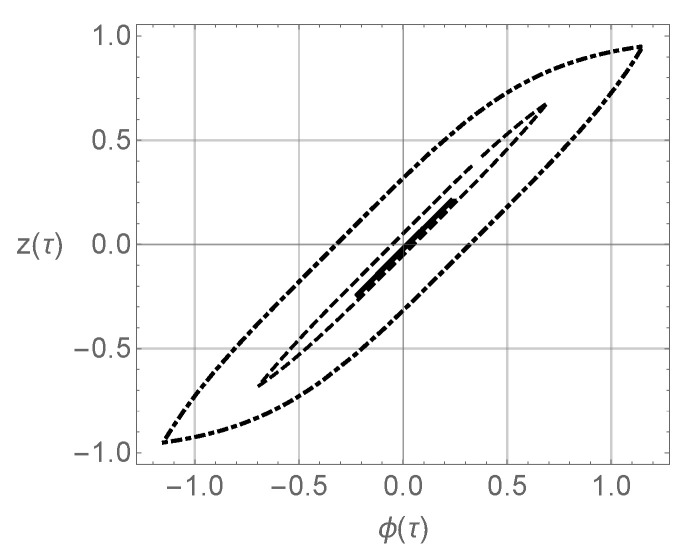
Hysteresis loop in coordinates (φ(t),z(t)) at K=5 (continuous line), K=15 (dashed line), K=25 (dash-dotted line), ω=1.2, Ω=5, and σ=1.5. Parameters of the Bouc–Wen model are A=1, β=0.1, γ=0.9, and n=3.

The hysteresis curve in coordinates (φ,z) is shown in Figure 15.

**Figure 16:**
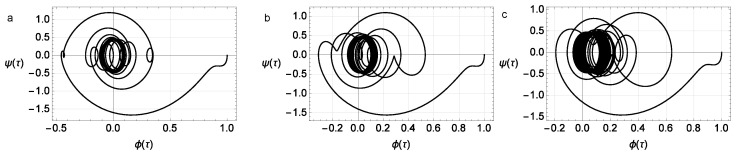
The phase portrait (φ(τ),v(τ)) at different values of the coefficient at the hysteresis term; ω=1.2, K=2, Ω=5, and σ=1.5. Parameters of the Bouc–Wen model: A=1, β=0.1, γ=0.9, and n=3, initial conditions φ(0)=1, v(0)=1, and (**a**) H=0.0, (**b**) H=0.5, or (**c**) H=1.

**Figure 17:**
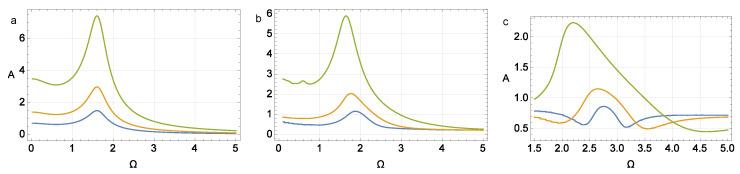
Amplitude–frequency characteristics for (42), ω=1.2, σ=1.5, and K=1 (blue line), K=2 (orange line), K=5 (green line). Parameters of the Bouc–Wen model: A=1, β=0.1, γ=0.9, and n=3, initial conditions φ(0)=1, v(0)=1, and (**a**) H=0, (**b**) H=1, or (**c**) H=5.

In Figure 16 phase portraits are given. Figure 17 shows a family of resonance curves for the mechanical part of system (42). Each of them exhibits the dependence of the amplitude of the solution on the frequency of the external periodic excitation Ω. We also show plots for various values of parameter *H*, which is responsible for the effect of the hysteresis block in the system. The results show that increasing this parameter significantly shifts the resonance frequency and also changes the shape and nature of the resonance curves depending on the amplitude of the forcing effect *K*. It is also worth noting a significant decrease in the amplitude of natural oscillations is observed on increasing the parameter *H* before the hysteresis block.

The energy of the external excitation u(τ)=Ksin(Ωτ) is equal to $E_{\mathrm{ext}}=\frac{{\dot{u}}^{2}}{2}$. Thus, the external excitation power can be calculated as:

(43) $$p_{\mathrm{ext}}=\frac{1}{T}\langle E_{\mathrm{ext}}\rangle =\frac{1}{T^{2}}\int_{0}^{T}\frac{{\dot{u}}^{2}}{2}d\tau =\frac{1}{8\pi }K^{2}\Omega^{3},$$

where T=2π/Ω is the period of steady-state oscillations.

Next, estimate the energy absorbed by the hysteresis block [66,67]. The equation connecting variables *z* and φ,

(44) $$\frac{z_{0}}{2}\left[{}_{2}F_{1}\left(1,\frac{1}{n},1+\frac{1}{n};\left(\beta -\gamma \right)z_{0}^{n}\right)+{}_{2}F_{1}\left(1,\frac{1}{n},1+\frac{1}{n};z_{0}^{n}\right)\right]=\frac{u_{\max}}{u_{y}},$$

has a single root, $z_{0}$. Here, $u_{\max}$ is the amplitude of the oscillatory process, $u_{y}=1$, and ${}_{2}F_{1}\left(\cdots \right)$ denotes the Gauss hypergeometric function ${}_{2}F_{1}\left(a,b,c;z\right)=$$1+\frac{ab}{c}\frac{z}{1!}+\frac{a(a+1)b(b+1)}{c(c+1)}\frac{z}{2!}+\cdots$

The hysteresis loop area is expressed through the root of Equation (44) as follows:

(45) $$\begin{array}{cc} S=2Au_{y}(2\frac{u_{\max}}{u_{y}}- & \left\{z_{0}\;{}_{2}F_{1}\left[1,\frac{1}{n},1+\frac{1}{n};\left(\beta -\gamma \right)z_{0}^{n}\right]+\frac{1}{2}z_{0}^{2}\;{}_{2}F_{1}\left[1,\frac{2}{n},1+\frac{2}{n};\left(\beta -\gamma \right)z_{a}^{n}\right]\right\}-\\ - & \left\{z_{0}\;{}_{2}F_{1}\left[1,\frac{1}{n},1+\frac{1}{n};z_{0}^{n}\right]-\frac{1}{2}z_{0}^{2}\;{}_{2}F_{1}\left[1,\frac{2}{n},1+\frac{2}{n};z_{0}^{n}\right]\right\}). \end{array}$$

The power absorbed by the hysteresis link is equal to $\frac{S}{T}$, where *S* is the area of the hysteresis loop in the space {(φ,z)}. As is mentioned above, the electrical power is equal to $p_{\mathrm{avr}}=\frac{1}{T}\int_{0}^{T}v^{2}\left(\tau \right)d\tau$.

Table 2 and Table 3 show the dependence of the power on the system parameters. The last column shows the relative power generated by the electrical part of the system with respect to the power of oscillations of the mechanical subsystem. These tables clearly show that the relative power depends both on the amplitude of the external excitation and on the value of the hysteresis term.

## 4. Conclusions

In the first part of this paper, we proposed a model for an energy harvester, the mechanical part of which is supposed to be an inverted pendulum. Within the proposed model, we investigated the stability of the linearized system. Particulary, it was proven that the stabilizing control of the pendulum, based on the principles of the feedback, enables the stabilization of the system. An important characteristic of the energy harvester is that the electrical power under the load is presented for the linearized system in a closed analytical form. During numerical experiments, we identified zones in the parameter space corresponding to the maximum power. Additionally, we identified the stability zones in the parameter space, the amplitude–frequency characteristics, as well as the dependence of the transmitted power on the coupling parameter.

The dynamics of the system in the case of the hysteresis friction in the transmission mechanical joint was given in the second part of this paper. The role of non-linear effects was shown within the design Preisach model and the phenomenological Bouc–Wen model.

## Figures and Tables

**Table 1:** Power of external excitation and transmitted power in the case of a hysteresis link within the Preisach model.

System Parameter Values	Power of External Excitation, $p_{\mathrm{ext}}$	Power Transmitted by the Hysteresis Link, S/T	Electrical Power, $p_{\mathrm{avr}}$	Ratio, $p_{\mathrm{avr}}/p_{\mathrm{ext}}$
K=1.0, Ω=1.8, ω=1.2, H=1.0	0.232048	0.193251	1.919358	8.27139
K=1.0, Ω=1.9, ω=1.2, H=1.0	0.272911	0.203482	1.366327	5.00650
K=1.0, Ω=2.0, ω=1.2, H=1.0	0.318310	0.133638	0.769240	2.41664
				
K=2.0, Ω=1.7, ω=1.2, H=1.0	0.781928	0.181108	7.845580	10.0336
K=2.0, Ω=1.8, ω=1.2, H=1.0	0.928192	0.196008	6.208168	6.68845
K=2.0, Ω=1.9, ω=1.2, H=1.0	1.091640	0.205938	4.121634	3.77563

**Table 2:** Power values at H=1.

System Parameter Values	Power of External Excitation, $p_{\mathrm{ext}}$	Power Transmitted by the Hysteresis Link, S/T	Electrical Power,$p_{\mathrm{avr}}$	Ratio, $p_{\mathrm{avr}}/p_{\mathrm{ext}}$
K=1, Ω=1.8, ω=1.2	0.232048	0.265033	0.0789995	0.340445
K=1, Ω=1.9, ω=1.2	0.272911	0.303263	0.0769097	0.281812
K=1, Ω=2.0, ω=1.2	0.318310	0.241098	0.0737095	0.231565
				
K=2, Ω=1.7, ω=1.2	0.781928	1.14649	0.349885	0.447464
K=2, Ω=1.8, ω=1.2	0.928192	1.24074	0.364847	0.393073
K=2, Ω=1.9, ω=1.2	1.091640	1.13051	0.359681	0.329486
				
K=5, Ω=1.5, ω=1.2	3.357174	4.0951	2.36116	0.703318
K=5, Ω=1.6, ω=1.2	4.074366	4.98311	2.60570	0.639535
K=5, Ω=1.7, ω=1.2	4.887051	5.21743	2.90695	0.594827
K=5, Ω=1.8, ω=1.2	5.801197	4.72778	3.13901	0.541097

**Table 3:** Power values at H=5.

System Parameter Values	Power of External Excitation, $p_{\mathrm{ext}}$	Power Transmitted by the Hysteresis Link, S/T	Electrical Power,$p_{\mathrm{avr}}$	Ratio, $p_{\mathrm{avr}}/p_{\mathrm{ext}}$
K=1, Ω=2.6, ω=1.2	0.699327	0.0749008	0.0670908	0.0959363
K=1, Ω=2.7, ω=1.2	0.783162	0.129436	0.0617794	0.0788846
K=1, Ω=2.8, ω=1.2	0.873442	0.134498	0.0598389	0.0685093
K=1, Ω=2.9, ω=1.2	0.970407	0.0966934	0.0615716	0.0634492
				
K=2, Ω=2.5, ω=1.2	2.486795	0.330892	0.269381	0.1083250
K=2, Ω=2.6, ω=1.2	2.797307	0.420344	0.26172	0.0935614
K=2, Ω=2.7, ω=1.2	3.132646	0.434923	0.249046	0.0795002
K=2, Ω=2.8, ω=1.2	3.493769	0.398539	0.237786	0.0680600
				
K=5, Ω=2, ω=1.2	7.957747	1.30756	0.981924	0.123392
K=5, Ω=2.1, ω=1.2	9.212087	1.665	1.02829	0.111624
K=5, Ω=2.2, ω=1.2	10.591761	1.82673	1.09544	0.103424
K=5, Ω=2.3, ω=1.2	12.102738	1.85318	1.17937	0.097446

## Data Availability

Not applicable.
